# Welcome to the supplement on measurement of healthy ageing

**DOI:** 10.1093/ageing/afad103

**Published:** 2023-10-30

**Authors:** Theresa Diaz, Anshu Banerjee

**Affiliations:** Department of Maternal, Newborn, Child and Adolescent Health and Ageing, World Health Organization, Geneva, Switzerland; Department of Maternal, Newborn, Child and Adolescent Health and Ageing, World Health Organization, Geneva, Switzerland

**Keywords:** healthy ageing, measurement, monitoring

## Key Points

- The biggest challenge to measure healthy ageing has been the plethora of ways to collect and analyse data.
- Given this challenge, it is necessary to make certain that measurement concepts are clearly defined and harmonised across surveys.
- This collection of articles systematically reviewed a vast number of surveys across several countries to identify best question items to use in surveys that measure healthy ageing.
- These reviews provide recommendations for items to include in standardized questionnaire and identified measurement areas in need of further research.

##  

For the first time in history, most people are living longer due to a large reduction in mortality at all ages, particularly amongst those who are older [1]. Recognising that people are living longer in 2020, Member States adopted the United Nations Decade of Healthy Ageing (2021–2030) at the Seventy-third World Health Assembly and Seventy-fifth United Nations General Assembly [2]. The Decade outlines four action areas and key enablers that provide strategic guidance for the Member States and other stakeholders working to achieve its goals [3]. One of these enablers is to strengthen data, research and innovation on healthy ageing to accelerate implementation.

One of the biggest challenges to strengthening data has been the plethora of ways to collect and analyse data for specific aspects of healthy ageing that is the intrinsic capacity (all the physical and mental capacities that a person can draw on), environments (where people live and conduct their lives) and functional ability (combines the intrinsic capacity of the individual, the environment a person lives in and how people interact with their environment). For each area, there are sub-domains that require several data items to calculate a score for that domain. For example, for functional ability, one domain is the ability to meet basic needs which includes multiple items to be measured such as adequate income, diet, housing, health and social services. Similarly, to intrinsic capacities for the domain of locomotor capacity, several items would need to be measured.

The baseline report of the UN Decade of Ageing attempted to bring together various nationally representative studies to analyse data on intrinsic capacity and functional ability to compare countries [4]. There were 42 country studies that had potentially comparable data on some measures of either the functional ability or intrinsic capacity; however, to compare countries a minimum of three comparable items was needed to estimate a score for each domain. This limited the number of domains that could be compared across countries. It was only possible to compare one domain of functional ability (i.e. ability to meet some basic needs) for 37 countries. For intrinsic capacity, only two domains (i.e. cognition and vitality) had a comparable measure across 36 countries. These were compared using the items of delayed word recall and hand grip to measure cognition and vitality, respectively.

Thus, it is necessary to make certain that measurement concepts are clearly defined and harmonised across surveys to ensure that each survey is measuring the same concepts and to allow comparability across countries. To better define concepts, experts in ageing along with the World Health Organisation (WHO) have developed definitions in vital capacity and locomotor capacity [5, 6] To harmonise measures, it is necessary to systematically examine how various surveys have measured the domains and items for intrinsic capacity and functional ability. This collection of articles systematically reviewed a vast number of surveys across several countries (Table 1). Although some of the reviews were able to identify measures or proxy measures that could be used others could not identify such measures.

**Table 1:** Articles conducting reviews of measures of domains of intrinsic capacity or functional ability

**Intrinsic capacity (e.g. visual acuity, grip strength, word recall)**	**Functional ability (e.g. getting dressed, managing money, making or keeping friends)**
Measures of locomotor capacity	Measures of mobility
Measures of vitality capacity	Measures of ability to learn, grow and make decision (functional ability set)
Measures of psychological capacity	Measures of older persons’ ability to build and maintain social relationships
Measures of cognitive capacity	Measures of ability to contribute
Measures of sensory functions	Ability to meet basic needs

This supplement also includes a development of the item pool for WHO ageism scale along with commentaries on the capability approach and Healthy Ageing framework, unified framework for the measurement of mobility in older persons and monitoring Healthy Ageing for the Next Decade: South Korea’s Perspective from representatives from Ministry of Health. In addition, short research report on effective coverage of long-term care services highlights the need to measure the impact of services on the person-centred outcomes.

The Technical Advisory Group on Measurement, Monitoring and Evaluation of UN Decade of Healthy Ageing (TAG4MHA) was established to provide advice on the measurement, monitoring and evaluation of the Decade and programmes related to the action areas [7]. One of the roles of the TAG4MHA is to make recommendations on what should be included in a standardised questionnaire on healthy ageing to ensure comparability of measures across countries and/or to define where additional research is needed. This supplement is a critical first step to provide the evidence for the TAG4MHA to recommend items to include in such a questionnaire and prioritise measurement areas in need of further research.

## Data Availability

It is publically available data from UNDESA. https://www.un.org/development/desa/pd/sites/www.un.org.development.desa.pd/files/wpp2022_summary_of_results.pdf.
